# Demirtas Two-Step Treatment Model in Hypofunctional-Obstructed Kidneys: An Extended Series and Long-Term Prospective Results

**DOI:** 10.7759/cureus.26128

**Published:** 2022-06-20

**Authors:** Abdullah Demirtaş, Abdullah Golbasi, Ahmet S Guleser, Gökhan Sönmez, Türev Demirtaş, Ahmet GÜR, Şevket T Tombul

**Affiliations:** 1 Urology, Erciyes University, Kayseri, TUR; 2 Urology, Nevsehir State Hospital, Nevşehir, TUR; 3 Urology, Yeni Yüzyıl University, Istanbul, TUR; 4 History of Medicine and Ethics, Erciyes University, Kayseri, TUR; 5 Urology, Kayseri City Hospital, Kayseri, TUR

**Keywords:** percutaneous nephrostomy, obstruction, non-function, kidney, hydronephrosis

## Abstract

Aim: This study aimed to present the short- and long-term results of Demirtas two-step treatment model in patients with obstructed-hydronephrotic kidneys functioning below 10%, and before deciding on nephrectomy, to evaluate whether this method can contribute to the accurate assessment.

Material and methods: This prospective study included patients with unilateral renal obstruction and renal parenchymal loss assessed by computed tomography and whose renal function was found to be below 10% in Tc-99m-dimercaptosuccinic acid. In the first step, percutaneous nephrostomy (PCN) was performed. Two weeks later, the second step was performed, in which patients were offered nephrectomy (if renal function was <10%) or etiology-based treatment (if renal function was ≥10%).

Results: Thirty-eight patients were included in the study, comprising 20 (52.6%) men and 18 (47.4%) women with a mean age of 51.3±16.8 years. Mean baseline renal function was 6.0%, which increased to 10.8% two weeks after PCN (p=0.001). Renal function increased to above 10% in 20 (52.6%) out of 38 patients. Of these, 17 patients underwent etiology-based treatment and baseline, two-week, and 12-month renal function levels were 7.0%, 17.5%, and 18.8%, respectively (p<0.001).

Conclusion: Demirtas two-step treatment model introduced in the present study can be recommended as a standard treatment modality in unilaterally obstructed kidneys functioning below 10% ability.

## Introduction

Urinary tract obstruction (UTO) is mostly caused by kidney stones, ureteral cancer, ureteropelvic junction (UPJ) obstruction, and ureterovesical junction (UVJ) obstruction. Loss of function is inevitable in kidneys exposed to prolonged UTO [1,2]. This duration of exposure varies according to the degree (complete vs. incomplete), etiology, and type of obstruction, and there is no clear data on the duration for this period [2,3].

In the current urological practice, nephrectomy is recommended for poor functioning kidneys below 10% depending on the clinical condition of the patient regardless of the characteristics of the obstruction, while obstruction-relieving treatment is recommended for kidneys with enough function [4,5]. Contrariwise, several recent studies have shown that kidneys functioning below 10% ability during obstruction may show a significant increase in function after the elimination of the obstruction and may exceed the 10% limit [6-12]. In our previous pilot study showed that approximately half of the patients with obstructed and non-functioning kidneys had a significant increase in renal function following the elimination of the obstruction [6].

In this study, we aimed to present the short- and long-term results of Demirtas two-step treatment model in a larger patient series, which was described in our previous study for obstructed kidneys functioning below 10%.

## Materials and methods

Trial summary

The aim of this clinical trial was to investigate whether loss of kidney function caused by ureteral obstruction (ureteral stone, UPJ stenosis, malignant stenosis, etc.), which resulted in parenchymal loss and was measured as non-functional in scintigraphy, could be reversed by diversion (i.e., by reducing intrarenal pressure).

General information about the project

A clinical trial has been registered for this study with the title "Effect of nephrostomy on relative function of obstructed kidney” on May 3, 2019, with the number - NCT03936673. Erciyes University Scientific Research Coordination Unit-BAP (TTU202110663) provided financial support to this project.

Rationale and background information

This study consists of two stages. In the first stage, the pilot study of the project was carried out. A total of 18 patients were included in the pilot study between 2016-2018, and early results were obtained regarding the efficacy of the nephrostomy tube in hydronephrotic and non-functioning kidneys [6]. Afterward, 20 more patients were added to the research population and the second step of the study was completed. At the same time, the long-term outcomes of the patients included in the pilot study were observed.

Study design and inclusion/exclusion criteria

Severe renal parenchymal loss was assessed by computed tomography (CT), and these patients underwent Tc-99m-dimercaptosuccinic acid (DMSA {DMSA-0}). Inclusion criteria were unilateral obstructed kidney with relative renal function (RRF) 10% or less, unilateral obstructed kidney with grade 2 or more hydroureteronephrosis, defined etiology for unilateral obstructed kidney estimated glomerular filtration rate (GFR) ≥30 mL/min, and approving the nephrostomy.

Of these, patients with renal function greater than 10% were excluded from the study. Other exclusion criteria included bilateral obstruction, solitary or transplanted kidneys, contraindications for percutaneous nephrostomy (PCN) (bleeding diathesis, renal mass), and signs of upper urinary tract infection associated with obstruction such as pyelonephritis and sepsis.

Protocol of the treatment model and follow-up

In this study, the two-step treatment model that was described in our previous study was administered [6]. In the first step, a PCN was placed in the obstructed kidney after performing biochemical tests (creatinine, blood urea nitrogen). Two weeks later, DMSA (DMSA-1) was taken for a second time and renal function was recorded. Both before and after PCN, GFR was calculated according to the short Modification of Diet in Renal Disease (MDRD) formula (GFR = 186 × {serum creatinine} - 1.154 × {Age} - 0.203 {if female} × 0.742 {if African American} × 1.212) [13]. In the second step, patients with a renal function above 10% in DMSA-1 underwent obstruction-relieving treatment (ureterorenoscopy, ureteroureterostomy, pyeloplasty) for the investigation of the etiology. Nephrectomy was performed in patients with renal function lower than 10% in DMSA-1. A control DMSA (DMSA-2) was taken at the end of post-operative month 12 for the investigation of the etiology in patients that received corrective treatment. In addition, some demographic and clinical characteristics including age, gender, body mass index (BMI), obstruction side, and etiology of obstruction were recorded for each patient. Patients with a follow-up period exceeding 12 months were included in routine follow-up protocols and their data was recorded in the hospital data system and followed up.

Percutaneous nephrostomy (PCN) placement

All the procedures were performed under local anesthesia and outpatient surgery setting with the patient placed in the prone position. Before the procedure, 1 g of ceftriaxone was administered intravenously. Under ultrasound guidance, the most suitable calyx was determined and entered using a 22-gauge Chiba needle. The mandrel of the needle was removed and urine output was observed. A guidewire was sent through the needle. Both cutaneous and subcutaneous tissues were resected approximately 5 mm with a no. 11 scalpel. Again, under ultrasound guidance, a tract was created with a 6-8-10 F dilator over the guidewire, and then an 8 Fr PCN tube was inserted into the collecting system through the tract. The tube was fixed to the skin with a 2/0 silk suture.

Safety considerations

In order to ensure the safety of the research, phone calls were made at 4-12 and 24 hours after the nephrostomy tube was applied to the patients. Patient follow-up forms were created to record adverse events and complications.

Expected outcomes of the study

It was expected that the patients included in the study were followed up for at least 12 months, the long-term follow-up results of the patients included in the pilot study were determined, and the compatibility between the pilot study and the main project results was evaluated.

Statistical analysis

Data were analyzed using SPSS for Windows version 22.0 (Armonk, NY: IBM Corp., USA). Normal distribution of continuous variables was assessed using the Kolmogorov-Smirnov test and histogram plots. Continuous variables with normal distribution were expressed as mean±standard deviation (SD), continuous variables with nonnormal distribution were expressed as median (first-third quartile), and categorical variables were expressed as percentages (%). In independent groups, continuous variables with normal distribution were compared using Student’s t-test and continuous variables with nonnormal distribution were compared using the Mann-Whitney U test. In dependent groups, binary comparisons of continuous variables with normal distribution were performed using dependent groups t-test and multiple comparisons were performed using repeated-measures ANOVA test. Categorical variables were compared using chi-square test. A p-value of <0.05 was considered significant.

## Results

Patient demographics

The study included 38 patients comprising 20 (52.6%) men and 18 (47.4%) women with a mean age of 51.3±16.8 years and a mean BMI of 28.4±5.6 kg/m^2^. UTO was present on the right side in 16 (42%) and in the left side in 22 (58%) patients. UTO was associated with ureteral stones in 17 (44.7%), ureteral stenosis in 20 (52.6%) (14 patients had UPJ obstruction, three patients had UVJ stenosis, and three patients had mid-ureteral stricture), and malignancy (cervical cancer) in one (2.6%) patient.

Short-term results

No significant difference was found between the groups with regard to serum creatinine, blood urea nitrogen (BUN), and GFR values ​​measured before and two weeks after PCN. However, renal function of 20 (52.6%) out of 38 patients increased to above 10% after PCN (Table 1).

**Table 1:** Comparison of pre- and post-PCN short-term kidney function tests of all patients (n=38). PCN: percutaneous nephrostomy; BUN: blood urea nitrogen; GFR: glomerular filtration rate

Variable	Pre-PCN	Two weeks after PCN	p-Value
Serum creatinine (mg/dL)	1.11	1.06	0.605
BUN (mg/dL)	29.03	20.94	0.503
GFR (mL/min/1.73 m^2^)	76.71	79.84	0.100
Renal function (%)	6.0	10.8	0.001

When compared according to etiologies including ureteral stones and ureteral stenosis, changes in DMSA-0 and DMSA-1 values ​​were statistically significant (6.59% vs. 11.74%, respectively) in patients with ureteral stenosis (n=20) (p<0.05). Although significant improvement was observed in DMSA-1 values ​​of 17 patients with ureteral stones, the difference was statistically insignificant (5.35% vs. 10.06%, respectively) (p=0.068).

Long-term results

An etiology-based treatment was performed in 17 out of 20 patients whose renal function increased to above 10% in DMSA-1, and the PCN tubes were removed after the treatment. The remaining three patients were excluded from follow-up, among whom one patient died from coronavirus disease 2019 (n=1) and the other two patients underwent nephrectomy. Table 2 presents a comparison of DMSA-0, DMSA-1, and DMSA-2 values ​​of 17 patients who underwent treatment, completed a 12-month follow-up, and presents the results of other renal function tests performed during this period.

**Table 2:** Evaluation of the renal functions of 17 patients who underwent etiology-based treatment and completed a 12-month follow-up. DMSA: Tc-99-m dimercaptosuccinic acid; BUN: blood urea nitrogen; GFR: glomerular filtration rate. *Statistically different. **Statistically similar. ***Statistically significant.

Variable	DMSA-0	DMSA-1	DMSA-2	p-Value
Serum creatinine (mg/dL)	1.10	1.12	1.05	0.268
BUN (mg/dL)	25.71	21.64	22.05	0.623
GFR (mL/min/1.73 m^2^)	77.71	83	82.1	0.316
Renal function (%)	7.0*	17.5**	18.8**	<0.001***

## Discussion

Kidneys are one of the most vital organs in the human body and thus need utmost protection [14]. Although there is no definite cut-off value, kidneys functioning below 10% ability are considered non-functional, and nephrectomy is often recommended for these patients although it is dependent on the clinical condition of the patient [5]. Our results indicated that following the insertion of PCN into the obstructed kidney, the obstruction was relieved and the renal function increased to above 10% in more than half of the patients. These findings implicate that when deciding on nephrectomy in hydronephrotic-obstructed kidneys, it would be a more appropriate approach to first reduce the stress on the kidney with a PCN and then assess renal function.

Literature indicated that the interest in the treatment of UTO started in early 2000s and the majority of the studies were conducted on pediatric patients. In 2001, Gupta et al., for the first time in the literature, reported that the renal function improved significantly following PCN insertion in 17 out of 20 children who had UPJ obstruction and kidneys functioning below 10% ability [7]. In a 2012 study, Bansal et al. evaluated pediatric patients with loss of renal function due to UPJ obstruction and reported that split renal function improved in 31 out of 35 children after pyeloplasty [15]. The same study, unlike our study, initially performed direct corrective surgery rather than PCN insertion. Based on our results, we suggest that the treatment administered by Bansal et al. could be a risky approach, particularly in patients with kidneys functioning below 10% ability since this method is likely to fail to improve renal function and thus may prevent nephrectomy.

In a recent study, Menon et al. evaluated pediatric patients aged below 12 years with UPJ obstruction and renal function below 20% ability who initially underwent PCN insertion and showed significant improvement in renal function. The authors proposed that PCN insertion should be the primary method in patients undergoing pyeloplasty/nephrectomy due to low renal function [9]. To our knowledge, the only study that examined adult patients with UPJ obstruction was published by Zhang et al. in 2015. The authors reported that the renal function increased to above 10% after PCN in 30 out of 53 patients and revealed that the success rate of this method was 56.6% [10]. These findings were supported by some case reports and case series [11,12,16]. Tonyali et al. reported recovery after nephrostomy in a case with hydronephrosis and hypofunctioning kidney [16]. The only study to argue otherwise was published by Kalra et al., which evaluated 17 patients with an average baseline renal function of 13% and reported that the renal function increased to 16% following PCN and improved in 12 (70.6%) patients. The authors also noted that split renal function only increased by 14% in patients with baseline renal function of less than 15% and suggested that direct nephrectomy should be offered to these patients [17]. The difference between the findings of that study and our study could be attributed to the fact that the patients included in that study had a baseline renal function above 10% and the others used a cut-off value of 15% rather than 10% as an indication for nephrectomy.

In our previous pilot study, we reported the early results of 18 patients [6]. In the present study, in contrast, the long-term results of 17 patients who completed a one-year follow-up period were analyzed and it was revealed that the increase in renal function detected after PCN was also maintained in the long term after etiology-based treatment (17.5% vs. 18.8%, p=0.965). In the literature, there are three noteworthy studies reporting on long-term results of patients [7,10,12]. The results of Zhang et al. showed that the rapid increase in renal function after PCN maintained a plateau over the six- and 12-month periods [10]. A study conducted on pediatric patients with UPJ obstruction reported that renal function was significantly preserved at the end of 12-month follow-up after pyeloplasty [12]. In that study, however, it is noteworthy that the number of patients with baseline renal function below 10% was remarkably low (n=4). Similar results were reported by Gupta et al. at the end of a mean follow-up period of 23 months [7]. Taken together, these findings implicate that the increase in renal function after PCN is permanent in the post-treatment period.

Almost all of the studies conducted on this subject have focused on UPJ obstruction that leads to prolonged renal obstruction. However, it was a question of interest whether our two-step treatment model would be effective in cases of acute obstruction such as those with urinary system stone disease. For this reason, we performed a subgroup analysis in the present study. The analysis indicated that the renal functions of 17 patients with ureteral stones increased from 5% to 10% on average after PCN, although the increase was statistically insignificant (p=0.068). This finding could be due to the small number of patients included in the study and we consider that successful results of our two-step treatment model will be reported in cases of acute obstruction in future studies. The only study in the literature that included patients with ureteral stones was published by Kalra et al., which did perform an evaluation since it had a small number of patients (n=7) and conducted no etiology-based analysis [17].

Our study has the following limitations: (1) the number of patients included in the study was remarkably small, (2) only DMSA was used for the evaluation of renal functions and scintigraphy (e.g., diethylenetriaminepentaacetic acid) was not employed for the evaluation of obstruction, and (3) daily urine output from the nephrostomy tube was not evaluated following PCN.

## Conclusions

Demirtas two-step treatment model introduced in the present study can be recommended as a standard diagnostic and treatment method for hypofunctional and obstructed kidneys. With this method, kidney function preservation can be ensured in more than half of patients with an initial indication of nephrectomy. Further prospective studies with larger patient series are needed to perform subgroup analyses according to etiological and demographic factors.
